# Functional intervention following cardiac surgery to prevent postoperative delirium in older patients (FEEL WELL study)

**DOI:** 10.1186/s40560-023-00711-1

**Published:** 2023-12-13

**Authors:** Tuğce Dinç Dogan, Vera Guttenthaler, Alexa Zimmermann, Andrea Kunsorg, Merve Özlem Dinç, Niko Knuelle, Jens-Christian Schewe, Maria Wittmann

**Affiliations:** 1 Department of Anaesthesia and Intensive Care Medicine, Istanbul University Istanbul Medical Faculty, Istanbul, Turkey; 2 Department of Anaesthesia and Intensive Care Medicine, University Hospital Bonn, Bonn, Germany; 3 University Bonn, Bonn, Germany; 4 Department of Anaesthesiology, Intensive Care Medicine and Pain Therapy, University Medical Centre Rostock, Rostock, Germany

**Keywords:** Cardiac surgery, Multisensory stimulation, Snoezelen, Older patients, Pain, Postoperative delirium

## Abstract

**Background:**

Postoperative delirium is a common complication in patients after cardiac surgery, especially in older patients, and can manifest as a disturbance of attention and consciousness. It can lead to increased postoperative morbidity, prolonged need for care, and mortality. The presented study investigates whether the occurrence of postoperative delirium after cardiac surgery can be prevented by a multisensory stimulation. It was conducted as a prospective, randomized, controlled, non-pharmacological intervention study in the years 2021 and 2022 at the University Hospital Bonn in Germany. A total of 186 patients over 65 years with elective cardiac surgery were enrolled. Patients were randomized either to the intervention or control group. In both groups, postoperative delirium was assessed with the 3-min diagnostic interview for confusion assessment method on the first 5 days after surgery and pain was assessed using the Numeric Rating Scale. Multisensory stimulation was performed 20 min a day for the first three postoperative days in the intervention group.

**Results:**

The incidence of postoperative delirium was 22.6% in the intervention group and 49.5% in the control group (*p* < 0.001). Duration of postoperative delirium was significantly shorter in the intervention group (*p* < 0.001). Stay in the intensive care unit was significantly longer in the control group (*p* = 0.006). In the regression model non-intervention, high pain scores, advanced age, and prolonged mechanical ventilation were associated with postoperative delirium (*p* = 0.007; *p* = 0.032; *p* = 0.006; *p* = 0.006, respectively).

**Conclusions:**

Results of the study imply that a multisensory stimulation done on the first 3 days after planned cardiac surgery can reduce the incidence and duration of postoperative delirium in older patients. Influence of the treatment on the incidence of delirium in other patient groups, the length of stay in the intensive care unit, and patients´ postoperative pain should be confirmed in further clinical studies.

*Trial registration*: DRKS, DRKS00026909. Registered 28 October 2021, Retrospectively registered, https://drks.de/search/de/trial/DRKS00026909.

## Background

Postoperative delirium (POD) is a common phenomenon, especially in older patients after cardiac surgery, and is associated with increased morbidity and mortality [1–3]. Many predisposing and precipitating factors for the occurrence of POD after cardiac surgery have been identified. Chen et al. identified in a meta-analysis aging, diabetes, preoperative depression, mild cognitive impairment, carotid artery stenosis, NYHA functional class III or IV, time of mechanical ventilation, and length of intensive care unit stay as risk factors [4]. POD can lead to prolonged need for care, contributes to increased healthcare costs [5, 6] and can be distressing to both the patients and their families [7].

The incidence of delirium after cardiac surgery is reported in the range of 4.5–54.9% [4]. In a prospective study of postoperative delirium conducted in the year 2019 at the University Hospital in Bonn, Germany, a POD incidence of 50.0% was found in patients undergoing elective cardiac surgery in a sample size of 254 patients with a mean age of 70.5 years [8].

Due to its negative association with mortality, morbidity, as well as prolonged hospitalization, prevention of POD is of high importance. Because of the high rate of polypharmacy in older patients [9], it might be better for them to receive an alternative treatment for the prevention of POD. One of the non-pharmacological methods preventing delirium could be the Multisensory stimulation (MSS) so called *Snoezelen*.

Snoezelen was first introduced in the 1970s as an intervention for people with learning disabilities, based on the rationale of reducing the adverse effects of sensory deprivation. Over the time this application has been extended to the care of older people with dementia as both groups share some common characteristics, such as reduced cognitive functions and diminished communicative ability [10].

It creates gentle stimulations and a relaxing atmosphere that helps to reduce agitation and anxiety.

The characteristics of the *Snoezelen* are: (a) visual, auditory, tactile, and olfactory stimulation in a room or environment using lights, music, aromas, and tactile objects; (b) individual and non-directive intervention in which participants choose the sensory stimuli; (c) use of non-sequential and non-standardized stimulus; (d) reduced cognitive requirements [11].

The frequency of sessions varies from 3 sessions total to daily sessions over a 15-month period [10, 12–16].

Behavioural research in Alzheimer's disease and other dementing disorders drew the conclusion, that a person with impaired consciousness is particularly vulnerable to environmental influences [17] and that older people with dementia experience intrapsychic discomfort, because the rates of sense-stimulating and sense-soothing activities are imbalanced [18].

This might appear in hospitalized patients after surgery, due to an unfamiliar and often noisy environment ([19, 20].

The aim of the study was to evaluate whether a 20-min multisensory stimulation on postoperative days 1–3 could be used as an easy performable, non-pharmacological means to reduce the incidence of POD in older patients after elective cardiac surgery.

## Methods

In this monocentric, prospective, randomized, controlled, non-pharmacological interventional study 237 patients that underwent elective cardiac surgery at the University Hospital Bonn from September 2021 until July 2022 were included. This study was carried out in accordance with the Helsinki Declaration. An ethics vote was provided by the Ethics Commitee of the Medical Faculty of the Rheinische Friedrich-Wilhelms-Universitaet Bonn (# 293/21). All consecutive patients older than 65 years with elective cardiac surgery were eligible if they were fluent in the German language, legally competent, and planned to be weaned from mechanical ventilation within 24 h after surgery (ınclusion criteria).

Exclusion criteria were emergency procedures, language barriers, documented severe psychiatric disorders or documented demantia. We excluded patients with a documented diagnosis of dementia, to minimize additional risk factors thought to be associated with delirium. To avoid the association between prolonged ventilation time and increased incidence of delirium, patients with an anticipated need for mechanical ventilation in the ICU for more than 24 h were also excluded. This patient group includes patients with severe respiratory comorbidities, that could lead to postoperative prolonged intubation, patients with preoperative respiratory failure or need for intubation and patients with a need for a left ventricular assist device (LVAD).

Written informed consent to the study was obtained from each patient before surgery. Then, patients were assigned to their respective groups (intervention group or control group) by lottery procedure.

Demographic and treatment data were collected by the study team from the anaesthesia protocols and the patient records. Patients were included consecutively over a period of 1 year and it is, therefore, anticipated, that both groups consisted of a comparable mix of cardiac surgical procedures.

Preoperative assessment, intraoperative handling, and postoperative treatment were performed the same in both groups according to the standard operating procedures of the University Hospital Bonn as only the study personnel knew about the actual group assignment of the patient.

Upon arrival in the introduction suite pulse oximetry, electrocardiogram (ECG), and a peripheral and arterial line were established. Subsequently, anaesthesia was induced using sufentanil, propofol (1–1.5 mg/kg), and rocuronium (0.5 mg/kg). Following intubation, anaesthesia was maintained using sevoflurane at BIS values between 40 and 60, ensuring appropriate anaesthesia level. Next a central line and sheath were inserted into the right jugular venae under ultrasound guidance. Oxygen concentration was adjusted to maintain SpO_2_ above 95%. Anaesthesia was maintained during surgery, including on-pump stages using sevoflurane and sufentanyl. At the end of surgery patients were transferred to ICU and extubated within 6 h after surgery according to current guidelines.

Postoperative treatment in the ICU was according to inhouse clinical standard and no intended differences between groups were made. In brief, patients were sedated with continuous infusions of propofol and sufentanil in the ICU during mechanical ventilation; neuromuscular blockade was not part of the regular regimen. Timing of extubation was left to the discretion of the attending physician who was blinded to patient study groups, according to the individual clinical course of the patient and whenever gas exchange was sufficient and patient was able to breath spontaneously and given hemodynamic stable situation, as well as when the patient reached normothermia. Additional clonidin was given in cases of shivering. Postoperative analgesia was regularly provided by piritramide as an individual bolus (2–5 mg) intravenously and additional metamizole intravenously as adjunctive analgesia when necessary or requested by the patient.

The day after surgery was defined as the first postoperative day. After the end of sedation and extubation with having a Richmond Agitation–Sedation Scale 0 or − 1 (RASS) [21], the 3-min diagnostic ınterview for confusion assessment method defined delirium (3D-CAM) was performed daily in the morning after routine medical treatments. Assessment continued from postoperative day 1 until day 5 and was scheduled to be finished before lunchtime. Testing was conducted by trained study personel in the ICU or the normal ward to detect a possible postoperative delirium in both groups. After the 5th day patients were no longer followed up as most incidences of POD happen in the first 5 days after surgery. It is assumed that, in accordance with the standard procedures of the ICU, no patients with delirium were discharged from ICU. The 3D-CAM can be completed in a median of 3 min, and has a sensitivity of 95% and specificity of 94% for detection of POD. The 3D-CAM is considered positive if acute onset or fluctuating course, inattention and either disorganized thinking or altered level of consciousness present according to the scoring system of the scale [22]. As the 3D-CAM test is not validated for patients on ICU, we set the sedation level for testing to a RASS score of 0 or − 1, which meant that patients were extubated and responsive. Acute pain was assessed postoperatively using the Numeric Rating Scale (NRS) at the time when patients were assessed for delirium [23]. Only the patients who were able to respond to the 3D-CAM test on a daily basis were asked to perform pain assessments with the NRS. The physicians who were responsible for the pain therapy were blinded to the *Snoezelen* treatment of the patient.

**Fig. 1:**
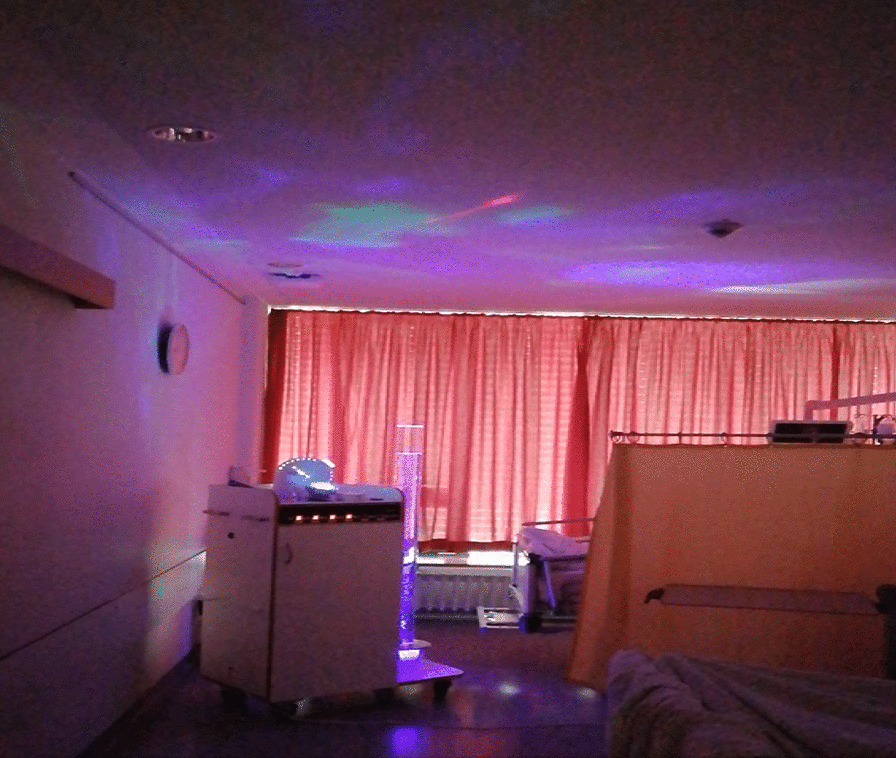
Multisensorial stimulation treatment

The intervention group received postoperative MSS treatment for three consecutive postoperative days. After lunchtime, patients, who were already extubated, were visited on ICU or the normal ward with a portable *Snoezelen* device with music system, projector, electronic candles, water column, scent machine and vibration pad on it. During the 20-min sessions, the room was darkened as much as possible and the patients listened to relaxing music according to their preference at a low volume and low pitch, and enjoyed the visuality that was created with light. The equipment consists of lighting effects such as electronic candles, illuminated water column and projector that can create different effects and images. The lights used were gentle, not flashing. All equipment was installed on a transportable device (Sinneswagen comfort + , Fa. Beluga, Germany). Depending on the patient's preference, aromatherapy scents and vibration cushion were also used. The practitioner who initiated the intervention left the patient alone for effective relaxation (Fig. 1).

The group of physicians and nurses who managed the care and treatment of the study patients, decisions on extubation, ward discharge, and analgesia applications were blinded to the study group.

Primary outcome was occurance of POD assessed in the ICU and the normal ward with the 3D-CAM. Secondary outcomes were length of stay in the ICU, length of stay in hospital, duration of delirium on postoperative days 1–5, and pain score after surgery assessed via NRS measuring the pain intensity from 0 to 10.

## Statistics

We expected a relative reduction of POD in the intervention group by 40% through the intervention [24, 25]. A total sample size of at least 186 patients was required to have a power of 80% to detect a decrease in the primary outcome from 50% in the control group (*n* = 93) to 30% in the experimental group (*n* = 93) with a two-sided significance level of 5%.

The exploratory statistical analysis was performed using the statistical programming environment R. Continuous variables are presented with median and interquartile range (IQR). A normal distribution of the continuous variables was not present. Categorical variables are shown as numbers and percentages (%). The differences between intervention and control group regarding the characteristics were analyzed using the non-parametric Wilcoxon rank-sum test for continuous variables and the Fisher’s exact test was computed to check for independence for categorical variables.

Logistic regression was performed to examine the effect of the intervention on POD development. POD entered the model as the binary outcome variable. Additional factors influencing POD were included as metric variables (ventilation time, NRS scores) or as categorical variables (age in increments of 5 years, The American Society of Anesthesiologists (ASA) classification and gender) and served as independent variables. The selected risk factors used in the logistic regression analysis represent accepted risk factors for development of postoperative delirium [5, 26, 27]. For better interpretability, (adjusted) odds ratios (OR) were generated via transformation from the regression coefficients and are reported with corresponding 95% confidence interval (CI).

## Results

This study was conducted and reported in conformance with the CONSORT guidelines for randomised trials [28]. 289 Patients were enrolled to the study between September, 2021 and July, 2022.

**Fig. 2:**
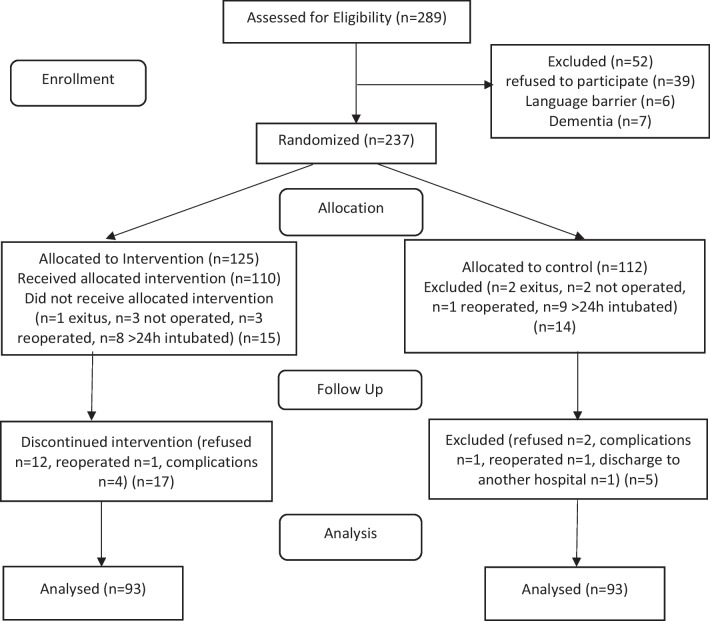
Flow diagram of included patients

Overall, 237 patients were randomised, of whom 125 were in the *Snoezelen* group, and 112 were in the control group. We included one new patient for every patient who dropped out of the study due to various reasons until we reached the number of 93 evaluable patients in each study group. 19 patients in the control group and 32 patients in the *Snoezelen* group were excluded from the study due to reasons, such as death, need for mechanical ventilation for > 24 h, cancellation of surgery, withdrawal of consent, and complications, such as bleeding requiring reoperation, acute cerebrovascular accident, and septic shock. 93 patients in each group were analysed (Fig. 2).

Patient characteristics and length of surgery were balanced among groups. The median age of the participating patients was 72 years. Age, proportion of male participants, ASA status, duration of surgery, grade of left ventricular ejection fraction, proportion of patients diagnosed with diabetes, and presence and severity of carotis artery stenosis did not differ significantly in the two study groups (Table 1). The Creatinine value and the estimated Glomerular Filtration Rate (eGFR) were comparable in both groups (Table 1). Of the 186 patients who participated in the study 137 were men. 98.3% of the patients were assigned to ASA physical status classification 3–4. No patient was in class ASA 1. 44.6% of all patients received preoperative oral midazolam for premedication (Table 1).

**Table 1:** Demographics

	Total (*n* = 186)	Group S (*n* = 93)	Group C (*n* = 93)	*p* value	Missing
Age (years)				0.306	0
Median (IQR)	72 (69–77)	73 (69–77)	71 (69–76)		
Male, *n* (%)	137 (73.7%)	69 (74.2%)	68 (73.1%)	1.000	0
Duration of surgery (min)				0.134	5
Median (IQR)	166 (123–235)	179 (131.25–245.75)	157 (121–222)		
ASA-scores, *n* (%)				0.704	2
ASA 1	0	0	0		
ASA 2	1 (0.5%)	0	1 (1.1%)		
ASA 3	113 (60.8%)	56 (60.2%)	57 (61.3%)		
ASA 4	70 (37.6%)	37 (39.8%)	33 (35.5%)		
NYHA				0.713	16
1	25 (13.4%)	14 (15.1%)	11 (11.8%)		
2	74 (39.8%)	40 (43.0%)	34 (36.6%)		
3	71 (38.2%)	34 (36.6%)	37 (39.8%)		
Diabetes				0.234	0
Yes	140 (75.3%)	74 (79.6%)	66 (71.0%)		
Left ventricular ejection fraction				0.921	8
Highly reduced	3 (1.6%)	2 (2.2%)	1 (1.1%)		
Moderately reduced	16 (8.6%)	9 (9.7%)	7 (7.5%)		
Slighty reduced	50 (26.9%)	25 (26.9%)	25 (26.9%)		
Normal	109 (58.6%)	53 (57.0%)	56 (60.2%)		
Creatinine (mg/dl)				0.115	0
Median (IQR)	0.97 0.84–1.16)	0.93 (0.81–1.16)	1.00 (0.88–1.16)		
eGFR (ml/min)				0.443	1
15–29	2 (1.1%)	0 (0.0%)	2 (2.2%)		
30–59	48 (25.8%)	23 (24.7%)	25 (26.9%)		
60–89	110 (59.1%)	54 (58.1%)	56 (60.2%)		
≥ 90	25 (13.4%)	15 (16.1%)	10 (10.8%)		
Carotid artery stenosis				0.321	0
High-grade stenosis or occlusion	3 (1.6%)	0 (0%)	3 (3.2%)		
Moderate stenosis up to 70% right and left	12 (6.5%)	6 (6.5%)	6 (6.5%)		
Light stenosis up to 50% right and left	11 (5.9%)	4 (4.3%)	7 (7.5%)		
No hemodynamically relevant sclerosis	160 (86.0%)	83 (89.2%)	77 (82.8%)		
Midazolam as preoperative medication, *n* (%)				1.000	1
Yes	83 (44.6%)	42 (45.2%)	41 (44.1%)		
Length of ICU stay (hours)				0.006*	9
Median (IQR)	23 (20–46)	23 (19–30.75)	26 (21–68)		
Length of hospital stay after surgery (days)				0.673	0
Median (IQR)	8 (7–11)	8 (7–11)	8 (7–10)		
Length of hospital stay total (days)				0.045*	0
Median (IQR)	11 (9–15)	12 (10–17)	11 (9–14)		
Duration of mechanical ventilation in ICU (hours)				0.014*	8
Median (IQR)	0 (0–7.75)	0 (0–5.75)	3 (0–8)		

*ASA* American Society of Anaesthesiologist classification, *NYHA* New York Heart Association classification, *ICU *ıntensive care unit, *IQR* ınterquartile range, *eGFR* estimated glomerular filtration rate, *Group S *ıntervention group, *Group C* control group

*Significant with *p* < 0.05

Patients stayed in the ICU for an average of 1–2 days after surgery. Duration of ICU stay differed significantly between the intervention and control group (*p* = 0.006) (Table 1). The median duration of mechanical ventilation after surgery in the ICU was significantly longer in the control group (*p* = 0.014) (Table 1). The median lenght of stay in hospital after surgery was 8 days and similar among both groups (*p* = 0.673) (Table 1). The total length of hospital stay was longer in the intervention group (*p* = 0.045) (Table 1).

The median duration of surgery in all patients was 166 min. Cardiac surgeries were classified as on-pump coronary artery bypass (CABG), off-pump coronary artery bypass, mitral valve surgery (MVR), aortic aneurysm surgery, aortic valve surgery (AVR) and complex surgeries (multiple valve replacement or combined bypass and valve replacement). There was no difference between the two groups regarding the types of operations (Table 2).

**Table 2:** Type of cardiac surgery

	Total (*n* = 186)	Group S (*n* = 93)	Group C (*n* = 93)	*p* value	Missing
Type of Surgery				1.000	0
CABG (on-pump)	61 (32.8%)	30 (32.3%)	31 (33.3%)		
CABG (off-pump)	33 (17.7%)	17 (18.3%)	16 (17.2%)		
MVR	30 (16.1%)	13 (14%)	17 (18.2%)		
Aneurysm	4 (2.2%)	3 (3.2%)	1 (1.0%)		
AVR	22 (11.8%)	11 (11.8%)	11 (11.8%)		
Complex	36 (19.4%)	19 (20.4%)	17 (18.3%)		

*CABG* coronary artery bypass graft surgery, *MVR* mitral valve replacement, *AVR *aortic valve replacement, *Complex* CABG + MVR/AVR or multiple cardiac valve replacement, *Group S *ıntervention group, *Group C* control group

*Significant with *p* < 0.05

Numeric Rating Scale (NRS) scores for pain intensity were similar between the two groups in the first 3 days (Table 3). In both groups, pain scores decreased day by day and from the first day on mean NRS-scores were equal or less than 5 points. The pain scores in the intervention group were significantly lower on days 4 and 5 (*p* = 0.022; *p* < 0.001, respectively) (Table 3). On day 4, the 15 missing NRS-values included 4 missing values from the intervention group and 11 values from the control group (Table 3).

**Table 3:** Numeric Rating Scale pain scores

	Total (*n* = 186)	Group S (*n* = 93)	Group C (*n* = 93)	*p* value	Missing
NRS score on day 1, median (IQR)	3 (1–5)	4 (1–5)	5 (0.3–5)	0.734	8
NRS score on day 2, median (IQR)	2 (0–5)	2 (0–4)	2 (0–6)	0.250	6
NRS score on day 3, median (IQR)	1 (0–4)	0 (0–3)	1 (0–5)	0.159	4
NRS score on day 4, median (IQR)	0 (0–3)	0 (0–2)	1 (0–4)	0.022*	15 (4:11)
NRS score on day 5, median (IQR)	0 (0–3)	0 (0–1)	1 (0–4)	< 0.001*	28 (13:15)

*Significant with *p* < 0.05

*NRS* Numeric Rating Scale, *Group S* ıntervention group, *Group C* control group, *IQR* ınterquartile range

On day 5, there were 13 values missing from the intervention group and 15 from the control group (Table 3).

The incidence of delirium on postoperative days 1–5 was 22.6% (21 of 93) in the *Snoezelen* group (group S) and 49.5% (46 of 93) in the control group (group C) (*p* < 0.001) (Table 4). The length of delirium was significantly shorter in the *Snoezelen* group (*p* < 0.001) (Table 4). No patient in this group had delirium lasting longer than 4 days.

**Table 4:** Duration of postoperative delirium

	Total (*n* = 186)	Group S (*n* = 93)	Group C (*n* = 93)	*p* value
Overall incidence of postoperative delirium, *n* (%)	67 (36.0%)	21 (22.6%)	46 (49.5%)	< 0.001*
Duration of delirium, *n* (%)				< 0.001*
POD on 1 day	25 (13.4%)	13 (14%)	12 (12.9%)	
POD on 2 days	11 (5.9%)	2 (2.2%)	9 (9.7%)	
POD on 3 days	13 (7.0%)	4 (4.3%)	9 (9.7%)	
POD on 4 days	9 (4.8%)	2 (2.2%)	7 (7.5%)	
POD on 5 days	9 (4.8%)	0 (0%)	9 (9.7%)	

*Group S *ıntervention group, *Group C* control group, *POD* postoperative delirium

*Significant with *p* < 0.05

Although cardiac and respiratory values of the patients were not documented during the treatment, relaxation and restfulness were reported by most of the patients.

Logistic regression was performed to examine the effect of the intervention on POD development. Included factors were intervention, pain score on day 1 (NRS score), gender, ventilation time, age in increments of 5 years and ASA classification, and served as independent variables. Our regression model revealed that non-intervention (adj. OR 2.67), NRS score on day 1 (adj. OR 1.16), age (adj. OR 1.62) and ventilation time (adj. OR 1.12) were significantly associated with POD (*p* = 0.007; *p* = 0.032; *p* = 0.006; *p* = 0.006, respectively) (Table 5).

**Table 5:** Regression model

	Adjusted odds ratio	95% confidence ınterval		*p* value
Ref. ıntervention	2.67	1.32	5.53	0.007*
NRS score on day 1	1.16	1.01	1.33	0.032*
Ventilation time	1.12	1.03	1.21	0.006*
ASA-Scores	1.06	0.50	2.21	0.879
Gender	1.12	0.50	2.44	0.785
Age (increments of 5 years)	1.62	1.16	2.33	0.006*

*NRS* Numeric Rating Scale, *ASA* American Society of Anaesthesiologist classification

*Significant with *p* < 0.05

## Discussion

In this prospective, randomized, controlled, non-pharmacological study, patients receiving a MSS intervention after elective cardiac surgery had a reduction of the delirium incidence by 54.4% which supports the hypothesis of the trial that the multisensory stimulation (so called *Snoezelen*) on postoperative days 1–3 may reduce the incidence of POD in this critically ill patient group. To the best of our knowledge, we did not encounter any studies in the literature that directly addressed the effect of *Snoezelen* treatment on POD. Some studies used music or bright light therapy, some of them used training of healthcare professionals on delirium awareness as intervention [5, 24, 25, 29–35]. The results were similar with ours. In the mHELP study Chen et al. also found a significant reduction of delirium incidence by 56% and a reduced hospital LOS of 2 days [36].

In our study the duration of delirium was shorter in the intervention group (*p* < 0.001). Milisen et al. could also shorten the duration of delirium in elderly hip-fracture patients by improving their nursing care and being aware of delirium in clinical practice [37].

The ASA scores were statistically similar between the groups but the percentage of ASA IV patients in the intervention group was higher. Although preoperative evaluation scores were worse, the length of ICU stay was shorter in our intervention group (*p* = 0.006). One component of this multifactorial situation may have been the positive effect of *Snoezelen* treatment on the recovery and well-being of patients. As shown in literature, non-pharmacological interventions, aimed at accelerating the recovery and well-being of patients, can shorten the duration of hospitalization which may also shorten the length of ICU stay as well [30, 33, 36].

The patient groups were determined by lottery in the preoperative period and the physicians making the extubation and treatment decision were blinded to the patient groups. However, the median duration of mechanical ventilation after surgery in the ICU was 0 h in the intervention group and 3 h in the control group, which was statistically significant (*p* = 0.014). Prolonged mechanical ventilation might be associated with prolonged intensive care admission and delirium [4, 27, 38]. This may be one of the reasons for prolonged stay in the ICU and increased delirium rates in the control group.

As a secondary outcome we investigated the length of stay in hospital after surgery, that was similar in both study groups (*p* = 0.0673). In older patients undergoing cardiac surgery, there are many factors other than delirium that influence length of hospitalization [39, 40]. Therefore, this multifactorial outcome may not be altered significantly by correction of a single parameter.

Nevertheless, although this treatment does not shorten the length of hospitalization, it reduces the length of stay in the ICU. Since prolonged length of stay in the ICU is associated with serious complications, such as resistant infections etc., it is important to shorten this period [41].

In both groups, pain scores decreased day by day and from the first day on median NRS-scores were equal or less than 5 points, indicating that effective analgesia was achieved in both groups. We questioned pain scores daily during delirium assessment after routine medical treatments and before *Snoezelen* treatment. The pain scores were not significantly lower in the intervention group on days 1–3, but significantly lower on days 4 and 5 (*p* = 0.022; *p* < 0.001, respectively). Although there were many missing values in the pain assessment on the fifth postoperative day, the distribution of these missing values was similar between the groups. Many studies have shown that *Snoezelen* treatment reduces agitation and anxiety in target groups and increases well-being [10–16, 18]. The reduction of the pain scores after 3 times of *Snoezelen* could lead to the conclusion, that the intervention could reduce anxiety and improve the well-being of the patient which could lead to a decrease of the pain scores. As shown in many studies, high pain scores are associated with a higher incidence of perioperative delirium [41, 42]. It can be said that the incidence of delirium could also be reduced by reducing pain. However, although statistically different, the pain scores were low in both groups and it can be said the difference was not clinically significant.

Postoperative delirium is significantly associated with a raised mortality in hospital, as well as an increase in cognitive deficits of the patient. Patients experiencing a POD have an augmented need for nursing home care following hospitalization which enhances health care system costs [43–45]. Some studies have shown that general health expenditures increase up to 2.5 times in patients who have delirium compared to a similar population who does not have delirium [46, 47]. Considering the disadvantages that arise to patients and the health care system with the incidence of a postoperative delirium an intervention, such as *Snoezelen*, that necessitates relatively little effort and easy available equipment, might be a way to reduce raising health care costs. It could be a very cost-effective and affordable expenditure, if it could reduce the costs due to prolonged stay in the ICU, hospitalization, increased caregiver expenses, incidence of cognitive dysfunction and complications, such as delirium-related falls. The treatment also requires practitioners and time. However, the fact that the practitioner is only needed for the setup of the device and is not actively involved, reduces the time requirement. In addition, the time needed for MSS preparation should in general be less than the time needed to care for a hyperdelirious patient.

In our regression model, we found that non-intervention, NRS score on day 1, age and ventilation time were significantly associated with delirium. In other words, while high pain scores, prolonged ventilation time and advanced age increased delirium, the applied non-pharmacological intervention contributed to the prevention of delirium. These results are supported by the literature reviews [2, 3, 5, 31, 35, 36].

## Limitations

Our study has some limitations:

1. Although the sample size is large enough to meet the primary outcome it was a monocentric study with a relatively small number of patients.
2. We are not able to prove in this study whether only *Snoezelen* treatment was effective or if there were other factors are involved. The reduction of POD incidence and duration could be due to the fact, that communication or interaction of any kind with the patient could prevent the occurrence of POD or shorten its length but it has to be considered, that all patients were visited during the testing period and communication was not limited to the intervention group. As only a limited number of concommittant parameters were assessed it could not be ruled out that other factors played a role in POD reduction, too.
3. In our study, we used the 3D-CAM test to diagnose delirium. However, although this test is valid, it is not used as the gold standard test and is not validated for its use in the ICU. In addition, as patients were only tested once a day some patients with delirium may have been missed due to its fluctuating course.
4. We excluded patients with a documented diagnosis of dementia, to minimize additional risk factors thought to be associated with delirium but we did not assess dementia in our patients ourselves. Therefore, we might have included patients with an already existing mild cognitive impairment that could influence the occurance of POD. However, as we randomized our patients into two groups, the probability to have included patients with cognitive impairment is the same for both groups.
5. We tried to keep the personnel on the wards blinded to the patient´s allocation to the respective study group. However, to minimize disruption during MSS treatment, the nurses were informed that the patient receives a study-related treatment without disclosing any study details and expected results. Therefore, the nurses were not completely blinded to the study.

## Conclusions

As a result of extended human lifespans and aging populations POD is a growing and challenging health care problem and prevention, early diagnosis and treatment are of great importance.

Results of the study conducted on patients who underwent cardiac surgery imply that an individually composed multisensory stimulation done for 20 min on the first 3 days after surgery might be able to reduce the incidence and duration of postoperative delirium in older patients. A positive influence of the treatment on the incidence of delirium in other patient groups, patient´s length of stay in the intensive care unit, and postoperative pain should be confirmed in further clinical studies.

## Data Availability

The data sets used and/or analysed during the current study are available from the corresponding author on reasonable request.

## Abbreviations

ICU: Intensive care unit
POD: Postoperative delirium
DSM-V: Diagnostic and Statistical Manual of Mental Disorders 5th edition
mHELP: Modified Hospital Elder Life Program
MSS: Multisensory stimulation
LOS: Length of stay
AD: Alzheimers’ disease
RASS Score: Richmond Agitation–Sedation Scale
3D-CAM: 3-Minute Diagnostic Interview for Confusion Assessment Method
CAM–ICU: Confusion assessment method for ICU
NRS: Numeric Rating Scale
ASA: American Society of Anaesthesiology classification

## References

[1] Salluh JIF, Wang H, Schneider EB, Nagaraja N, Yenokyan G, Damluji A (2015). Outcome of delirium in critically ill patients: systematic review and meta-analysis. BMJ.

[2] Oh ST, Park JY (2019). Postoperative delirium. Korean J Anesthesiol.

[3] Kotfis K, Szylińska A, Listewnik M, Strzelbicka M, Brykczyński M, Rotter I (2018). Early delirium after cardiac surgery: an analysis of incidence and risk factors in elderly (≥ 65 years) and very elderly (≥ 80 years) patients. Clin Interv Aging.

[4] Chen H, Mo L, Hu H, Ou Y, Luo J (2021). Risk factors of postoperative delirium after cardiac surgery: a meta-analysis. J Cardiothorac Surg.

[5] Inouye SK, Westendorp RGJ, Saczynski JS (2014). Delirium in elderly people. Lancet.

[6] WHO European health information at your fingertips. [Internet]. [cited 2023 Aug 15]. Available from: https://gateway.euro.who.int/en/datasets/european-mortality-database/#all-causes.

[7] Morita T, Akechi T, Ikenaga M, Inoue S, Kohara H, Matsubara T (2007). Terminal delirium: recommendations from bereaved families’ experiences. J Pain Symptom Manage.

[8] Kirfel A, Menzenbach J, Guttenthaler V, Feggeler J, Mayr A, Coburn M (2021). Postoperative delirium after cardiac surgery of elderly patients as an independent risk factor for prolonged length of stay in intensive care unit and in hospital. Aging Clin Exp Res.

[9] Pazan F, Wehling M (2021). Polypharmacy in older adults: a narrative review of definitions, epidemiology and consequences. Eur Geriatr Med.

[10] Chung JC, Lai CK, Chung PM, French HP (2002). Snoezelen for dementia. Cochrane Database Syst Rev.

[11] Baker R, Bell S, Baker E, Holloway J, Pearce R, Dowling Z (2001). A randomized controlled trial of the effects of multi-sensory stimulation (MSS) for people with dementia. Br J Clin Psychol.

[12] Sánchez A, Millán-Calenti JC, Lorenzo-López L, Maseda A (2013). Multisensory stimulation for people with dementia: a review of the literature. Am J Alzheimers Dis Other Demen.

[13] Sánchez A, Maseda A, Marante-Moar MP, de Labra C, Lorenzo-López L, Millán-Calenti JC (2016). Comparing the effects of multisensory stimulation and individualized music sessions on elderly people with severe dementia: a randomized controlled trial. J Alzheimer’s Dis.

[14] Milev RV, Kellar T, McLean M, Mileva V, Luthra V, Thompson S (2008). Multisensory stimulation for elderly with dementia: a 24-week single-blind randomized controlled pilot study. Am J Alzheimers Dis Other Demen.

[15] Van Weert JCM, Van Dulmen AM, Spreeuwenberg PMM, Ribbe MW, Bensing JM (2005). Behavioral and mood effects of snoezelen integrated into 24-hour dementia care. J Am Geriatr Soc.

[16] Strøm BS, Ytrehus S, Grov EK (2016). Sensory stimulation for persons with dementia: a review of the literature. J Clin Nurs.

[17] Kovach CR (2000). Sensoristasis and imbalance in persons with dementia. J Nurs Scholarsh.

[18] Pinto JO, Dores AR, Geraldo A, Peixoto B, Barbosa F (2020). Sensory stimulation programs in dementia: a systematic review of methods and effectiveness. Expert Rev Neurother.

[19] Fischer ME, Cruickshanks KJ, Schubert CR, Pinto AA, Carlsson CM, Klein BE (2016). Age-related sensory impairments and risk of cognitive impairment. J Am Geriatr Soc.

[20] Yu R, Woo J (2019). Cognitive assessment of older people: do sensory function and frailty matter?. Int J Environ Res Public Health.

[21] Ely EW, Truman B, Shintani A, Thomason JWW, Wheeler AP, Gordon S (2003). Monitoring sedation status over time in ICU patients: reliability and validity of the Richmond Agitation-Sedation Scale (RASS). JAMA.

[22] Marcantonio ER, Ngo LH, O’Connor M, Jones RN, Crane PK, Metzger ED (2014). 3D-CAM: derivation and validation of a 3-minute diagnostic interview for CAM-defined delirium. Ann Intern Med.

[23] Alghadir AH, Anwer S, Iqbal A, Iqbal ZA (2018). Test–retest reliability, validity, and minimum detectable change of visual analog, numerical rating, and verbal rating scales for measurement of osteoarthritic knee pain. J Pain Res.

[24] Inouye SK, Bogardus ST, Charpentier PA, Leo-Summers L, Acampora D, Holford TR (1999). A multicomponent intervention to prevent delirium in hospitalized older patients. N Eng J Med.

[25] Marcantonio ER, Flacker JM, Wright RJ, Resnick NM (2001). Reducing delirium after hip fracture: a randomized trial. J Am Geriatr Soc.

[26] Scholz AFM, Oldroyd C, McCarthy K, Quinn TJ, Hewitt J (2016). Systematic review and meta-analysis of risk factors for postoperative delirium among older patients undergoing gastrointestinal surgery. Br J Surg.

[27] Burkhart CS, Dell-Kuster S, Gamberini M, Moeckli A, Grapow M, Filipovic M (2010). Modifiable and nonmodifiable risk factors for postoperative delirium after cardiac surgery with cardiopulmonary bypass. J Cardiothorac Vasc Anesth.

[28] Schulz KF, Altman DG, Moher D (2010). CONSORT 2010 Statement: updated guidelines for reporting parallel group randomised trials. BMJ.

[29] Hshieh TT, Yue J, Oh E, Puelle M, Dowal S, Travison T (2015). Effectiveness of multicomponent nonpharmacological delirium interventions: a meta-analysis. JAMA Intern Med.

[30] Chen CCH, Lin MT, Tien YW, Yen CJ, Huang GH, Inouye SK (2011). Modified hospital elder life program: effects on abdominal surgery patients. J Am Coll Surg.

[31] Caplan GA, Harper EL (2007). Recruitment of volunteers to improve vitality in the elderly: the REVIVE study. Intern Med J.

[32] McCaffrey R, Locsin R (2004). The effect of music listening on acute confusion and delirium in elders undergoing elective hip and knee surgery. J Clin Nurs.

[33] Lundström M, Edlund A, Karlsson S, Brännström B, Bucht G, Gustafson Y (2005). A multifactorial intervention program reduces the duration of delirium, length of hospitalization, and mortality in delirious patients. J Am Geriatr Soc.

[34] McCaffrey R (2009). The effect of music on acute confusion in older adults after hip or knee surgery. Appl Nurs Res.

[35] Ono H, Taguchi T, Kido Y, Fujino Y, Doki Y (2011). The usefulness of bright light therapy for patients after oesophagectomy. Intensive Crit Care Nurs.

[36] Chen CCH, Li HC, Liang JT, Lai IR, Purnomo JDT, Yang YT (2017). Effect of a modified hospital elder life program on delirium and length of hospital stay in patients undergoing abdominal surgery. JAMA Surg.

[37] Milisen K, Foreman MD, Abraham IL, De Geest S, Godderis J, Vandermeulen E (2001). A nurse-led interdisciplinary intervention program for delirium in elderly hip-fracture patients. J Am Geriatr Soc.

[38] Trudzinski FC, Neetz B, Bornitz F, Müller M, Weis A, Kronsteiner D (2022). Risk factors for prolonged mechanical ventilation and weaning failure: a systematic review. Respiration.

[39] Engelman DT, Ben Ali W, Williams JB, Perrault LP, Reddy VS, Arora RC (2019). Guidelines for perioperative care in cardiac surgery: enhanced recovery after surgery society recommendations. JAMA Surg.

[40] Waite I, Deshpande R, Baghai M, Massey T, Wendler O, Greenwood S (2017). Home-based preoperative rehabilitation (prehab) to improve physical function and reduce hospital length of stay for frail patients undergoing coronary artery bypass graft and valve surgery. J Cardiothorac Surg.

[41] Jin Z, Hu J, Ma D (2020). Postoperative delirium: perioperative assessment, risk reduction, and management. Br J Anaesth.

[42] Bilge EÜ, Kaya M, Şenel GÖ, Ünver S (2015). The incidence of delirium at the postoperative intensive care unit in adult patients. Turk J Anaesthesiol Reanim.

[43] Adamis D, Treloar A, Martin FC, Macdonald AJD (2006). Recovery and outcome of delirium in elderly medical inpatients. Arch Gerontol Geriatr.

[44] Inouye SK (2006). Delirium in older persons. N Engl J Med.

[45] Grover S, Shah R (2011). Distress due to delirium experience. Gen Hosp Psychiatry.

[46] Caplan GA, Teodorczuk A, Streatfeild J, Agar MR (2020). The financial and social costs of delirium. Eur Geriatr Med.

[47] Leslie DL, Marcantonio ER, Zhang Y, Leo-Summers L, Inouye SK (2008). One-year health care costs associated with delirium in the elderly. Arch Intern Med.

